# Positive Correlation of Serum N-Acetyl-*β*-hexosaminidase with Markers of Atherosclerosis in Diabetes Type 2 Patients with Mild Symptoms of Depression and Anxiety

**DOI:** 10.1155/2018/1760592

**Published:** 2018-06-20

**Authors:** Sylwia Chojnowska, Iwona Cabaj-Wiater, Aleksandra Mikulska-Baran, Beata Zalewska-Szajda, Napoleon Waszkiewicz

**Affiliations:** ^1^Faculty of Health Sciences, Lomza State University of Applied Sciences, Łomża, Poland; ^2^MEDI-System, Warsaw, Poland; ^3^Department of Pediatrics, Garwolin District Hospital, Garwolin, Poland; ^4^Department of Imaging Diagnostic, Children University Hospital, Białystok, Poland; ^5^Department of Psychiatry, Medical University of Białystok, Białystok, Poland

## Abstract

**Background:**

Analysis of the correlation between diabetes type 2 (DT2) and serum N-acetyl-*β*-hexosaminidase (HEX) activity with parameters of fat metabolism and symptoms of anxiety and depression.

**Material and Method:**

The study was performed using a random sample of 40 DT2 patients (22 women and 18 men) between the ages of 43 and 71 (median 59) and 40 control persons (28 women and 12 men) between the ages of 18 and 64 (median 46). The activity of HEX was determined by a colorimetric method. The activity of the serum exoglycosidase was expressed in pkat/mL. Each participant underwent Hamilton tests, to evaluate level of anxiety and depression. Additionally, the HEX activity and concentration of particular lipidograms were monitored using a blood sample from each participant.

**Results:**

In DT2 patients, a significant positive correlation was found between serum HEX activity and the concentration of serum cholesterol LDL fractions, triacylglycerols (TAG), and Castelligro atherogenic indexes. A significantly increased level of anxiety and depression in comparison to the control group was found as well.

**Conclusion:**

Serum HEX activity in DT2 patients is a better marker of atherosclerosis than serum total cholesterol level in persons with mild symptoms of depression and anxiety. In DT2 patients, a routine testing of anxiety and depression is recommended. Early detection of these disorders creates the possibility for treatment, an improvement in a patient's quality of life, and the overall longevity of DT2 patients.

## 1. Introduction

Defects in pancreatic insulin secretion [1] and/or defects in insulin action result in DT2 (constituting 90% of all diabetes cases) [2]. Patients with DT2 exhibit hyperglycemia and an increase in free radical concentration that disturbs many organs [3], particularly the eyes, kidneys, nerves, heart, and blood vessels [4, 5]. According to the World Health Organization, a yearly increase in the number of diabetic patients has reached 7.5 million [6]. Such high increase in morbidity designates diabetes as one of the most important chronic noninfectious diseases of the 21st century. Some DT2 risk factors include genetic predispositions, environmental influences as viral infections (enteroviruses, mumps, rubella, and HCV), unsuitable diet, exposition on toxic substances, or vitamin D deficiency [7, 8]. There is a consensus that inflammation often accompanies the onset of DT2; however, there is still debate as to whether DT2 results from this inflammation [8–11]. Lack of insulin action and oxidative stress changes the composition and action of many tissues and organs, which results in the development of depression among DT2 patients [12, 13] and DT2 in people suffering from depression [14, 15]. Roughly 11% of diabetics were diagnosed with depression, and 31% of diabetics reported some symptoms of depression [16]. In some cases of DT2, episodes related to anxiety were detected [17]. Unfortunately, DT2 patients are very rarely tested for depression and/or anxiety, probably because the doctors have insufficient knowledge. Additionally, there are only few easy tests available for the diagnosis of anxiety and/or depression in DT2 patients. Anxiety and/or depression in diabetic patients results in a lack of self-control regarding health, which makes it difficult for metabolic compensation of diabetes. Noncompensated diabetes intensifies the inflammatory state, damages vascular endothelium, accelerates development of diabetic atherosclerosis, and increases nephropathies and neuropathies [18]. An intensified inflammatory state is marked by an increase in activity of lysosomal exoglycosidases in tissues and body fluids [19]. Lysosomal exoglycosidases are enzymes that degrade glycoconjugates (glycoproteins, glycolipids, and proteoglycans). The most active lysosomal exoglycosidase is N-acetyl-*β*-hexosaminidase (HEX). HEX releases N-acetylglucosamine and N-acetylgalactosamine from the nonreducing ends of glycoconjugates oligosaccharide chains [20]. Serum and urinary HEX activity is sensitive and a specific marker of alcohol dependence [21] and colon cancer [22]. In the saliva of diabetes type 1 and diabetes type 2 patients [23] and diabetic pregnant women [24], a significant increase in HEX activity was found in comparison to healthy persons.

The aims of the present paper are to evaluate anxiety/depression in DT2 patients and to determine the relationship between serum parameters of atherosclerosis (cholesterol, HDL cholesterol, LDL cholesterol, and TAG) and serum HEX activity.

## 2. Material and Methods

### 2.1. Ethics

The research was approved by the Bioethics Committee at the Medical University of Białystok, Poland (protocol number: R-I-002/256/2015).

### 2.2. Patients

The study group consisted of 40 persons (18 ♂/22 ♀), 43–71 years old (58.4 ± 7.1 median of years) with diagnosed DT2. The control group consisted of 40 healthy volunteers (12 ♂/28 ♀), 18–64 years old (44.6 ± 12.8 median of years). Volunteers were healthy persons who routinely visited family doctors for prophylactic investigation.

#### 2.2.1. Methods

*(1) Evaluation of Anxiety and Depression*. In a sunny, warm, and quiet room, the anamnesis of each participant was taken for 20–30 minutes, and their level of anxiety and depression was determined. Anxiety was evaluated with a Hamilton Anxiety Rating Scale—HARS [25, 26], and depression was evaluated with a seventeen-item Hamilton Depression Rating Scale—HDRS [27, 28], which does not contain 4 items from a 21-item HDRS: diurnal variation, depersonalization/derealization, paranoid symptoms, and obsessional symptoms.

*(2) Blood Serum*. The blood was collected from the cubital vein of each participant into two centrifuge tubes. Tubes were left (30–60 min) at a laboratory temperature and then centrifuged (10 min, 4000 rpm, and +4°C) in a MPW-350R centrifuge (MPW Medical Instruments, Warsaw, Poland). The serum received in this way was transferred to plastic tubes and stored (−80°C) until determinations.

*(3) Determination of N-Acetyl-β-hexosaminidase*. The substrate for the determination of the serum HEX activity was 4-nitrophenyl-N-acetyl-*β*-glucosaminide (Sigma-Aldrich, St. Louis, USA), and the calibrant was 0.25 mmol/L 4-nitrophenol (Sigma-Aldrich, St. Louis, USA). Determination of the HEX activity in serum was performed in duplicates by the colorimetric method designed by Marciniak et al. [29]. This method is as follows: to one well of microplate (96 well U Transparent, Greiner Bio-One, Germany) was added: 10 *μ*L of serum, 40 *μ*L of 0.1 mol/L McIlvaine phosphate-citrate buffer, pH 4.7, and 30 *μ*L of 6.7 mmol/L of 4-nitrophenyl-N-acetyl-*β*-glucosaminide. Then, the microplate contents were mixed and incubated at 37°C for 60 min with mixing in a shaker with an incubator to microplate series DTS (ELMI Ltd. laboratory equipment, Latvia). Enzymatic reaction was terminated by adding 200 *μ*L of a 0.2 mol/L borate buffer at pH 9.8. The amount of released 4-nitrophenol was measured at 410 nm in a colorimeter (Infinite® 200 PRO, TECAN, Switzerland). HEX activity was expressed in pkat/mL of serum.

*(4) Determination of Serum Lipidogram*. The components of serum lipidogram, which are total serum cholesterol, HDL cholesterol, LDL cholesterol, and triacylglycerols, were determined with laboratory analyzer cobas 6000 (Roche, Switzerland) applying a reagent set that was supplied by the analyzer producer (Roche, Switzerland).

*(5) Castelligro Atherogenic Index*. Castelligro atherogenic index evaluates atheromatosis risk and is based on relations between total cholesterol and HDL fraction.

### 2.3. Statistical Analysis

The results were analyzed using Statistica 10 (StatSoft, Poland)—Mann–Whitney *U* test and Spearman's correlation. Statistical significance was set at ^∗^*p* < 0.05; ^∗∗^*p* < 0.01; ^∗∗∗^*p* < 0.001.

## 3. Results

**Figure 1:**
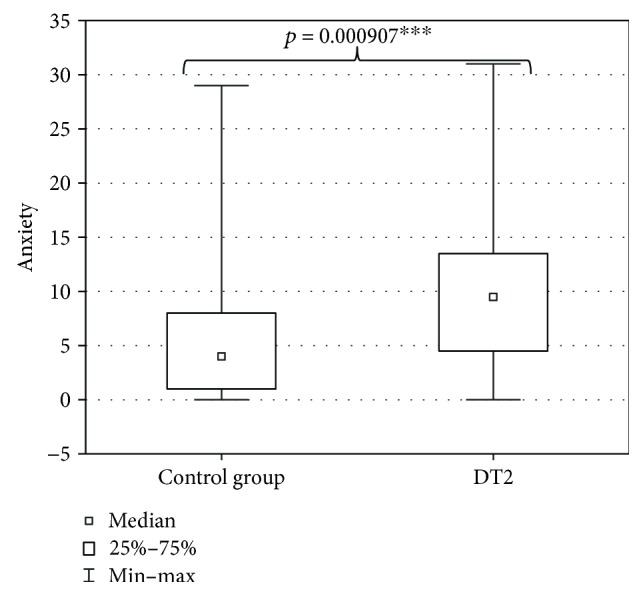
Anxiety in DT2 patients (statistical significance: ^∗∗∗^*p* < 0.001).

Evaluation with HARS of the DT2 patients and persons of the control group did not prove anxiety in both groups; however, significantly higher predisposition to anxiety was found in DT2 patients (9.5 ± 7.4 points) in comparison to persons of the control group (4 ± 7.2 points) (Figure 1).

**Figure 2:**
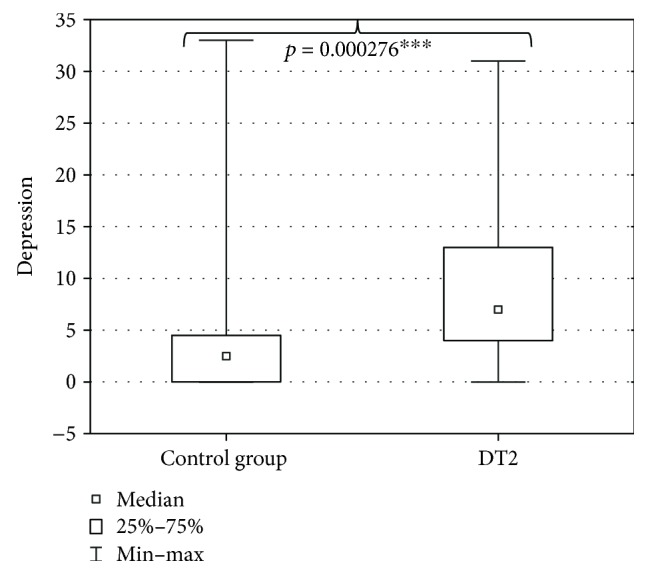
Depression in DT2 patients (statistical significance: ^∗∗∗^*p* < 0.001).

**Figure 3:**
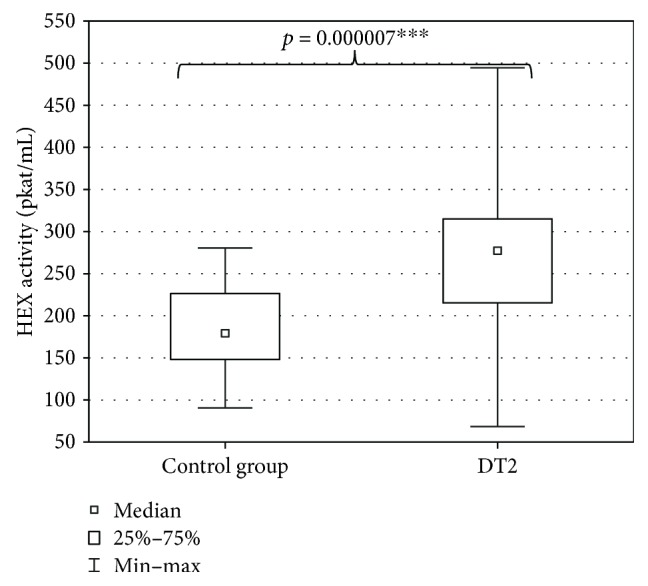
Activity of the serum HEX in DT2 patients (statistical significance: ^∗∗∗^*p* < 0.001).

**Figure 4:**
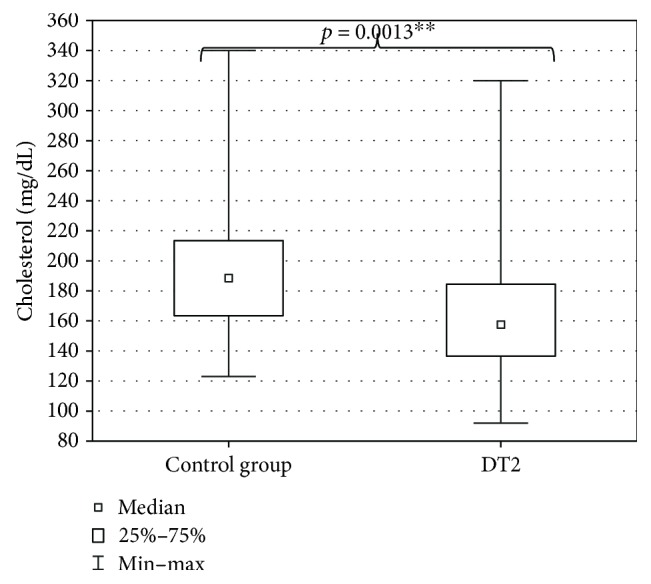
Serum cholesterol in DT2 patients (statistical significance: ^∗∗^*p* < 0.01).

**Figure 5:**
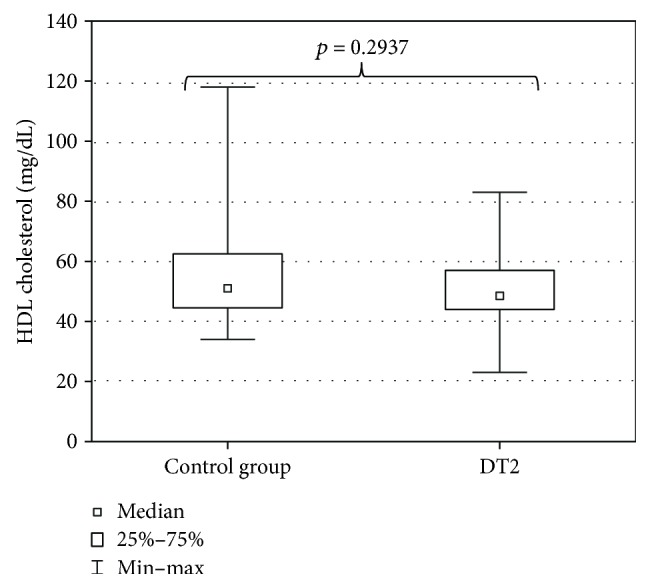
Serum HDL cholesterol in DT2 patients (statistical significance: *p* < 0.05).

**Figure 6:**
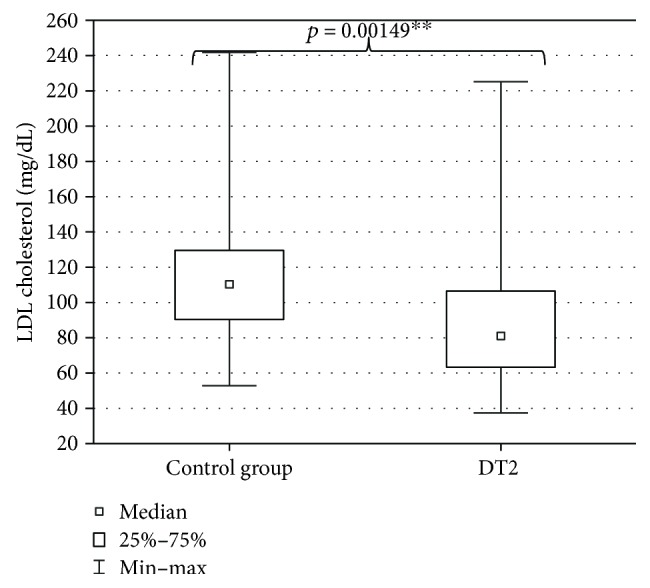
Serum LDL cholesterol in DT2 patients (statistical significance: ^∗∗^*p* < 0.01).

**Figure 7:**
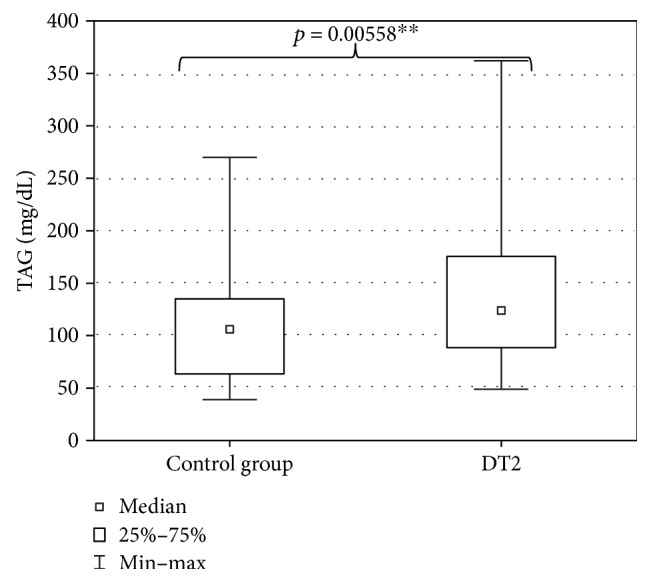
Serum triacylglycerols (TAG) in DT2 patients (statistical significance: ^∗∗^*p* < 0.01).

The evaluation of DT2 patients and persons of the control group with a shortened HDRS indicated mild depression (7 ± 7.4 points) in DT2 patients and absence of depression in the persons of the control group (2.5 ± 6.6 points). The level of depression in DT2 patients was significantly higher than in the control group (Figure 2). Serum HEX activity in DT2 patients amounted to 270.85 ± 95.58 pkat/mL and was significantly higher than HEX activity in the serum of the control group (188.04 ± 53.86 pkat/mL) (Figure 3). Concentration of serum total cholesterol in DT2 patients amounted to 157.5 ± 45 mg/dL and has a tendency to decrease in comparison to the concentration in the serum of the control group (188.5 ± 41.9 mg/dL) (Figure 4). Serum HDL cholesterol in DT2 patients (48.5 ± 12.3 mg/dL) has a lower tendency to decrease in comparison to the control group (51 ± 17.4 mg/dL) (Figure 5). Concentration of serum LDL cholesterol in DT2 patients amounted to 81 ± 38 mg/dL and was significantly lower than the LDL cholesterol concentration in the serum of the control persons (106 ± 60.5 mg/dL) (Figure 6). Concentration of TAG in the serum of the DT2 patients (124 ± 68 mg/dL) was significantly higher than the TAG concentration in the serum of the control group (106 ± 60.5 mg/dL) (Figure 7).

In DT2 patients, there were no significant correlations between level of anxiety, depression, serum total cholesterol, HDL cholesterol, and serum HEX activity (Table 1). In DT2 patients, the significant positive correlations were found between serum HEX activity and TAG concentration (*r* = 0.32, *p* = 0.046^∗^), concentration of LDL cholesterol (*r* = 0.37, *p* = 0.02^∗^), and values of Castelligro atherogenic index (*r* = 0.41, *p* = 0.008^∗∗^) (Table 1).

## 4. Discussion

**Figure 8:**
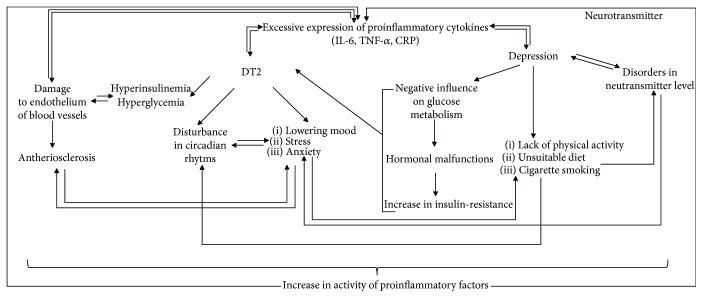
Relations between DT2, atherosclerosis, and depression based on [37–40].

Diagnosis of noninfectious chronic disease is usually connected with patient stress and accompanied by an increase in anxiety and appearance of depression. Additionally, DT2 patients are forced to rearrange their current agenda, which creates additional emotional tension. Unfortunately, in the case of DT2, the prognosis concerning quality of life and longevity is not optimistic. Literature data suggested that it is difficult to arbitrate if anxiety and depression favour development of DT2 or if DT2 patients have increased tendency to develop anxiety and depression [12, 13]. Depressed people reveal some risk factors of DT2 and atheromatosis more frequently, for example, low physical activity, obesity, and cigarette smoking, than general population [30]. It was reported that depression-related disorders increased the secretion of hormones regulating glucose metabolism, disturbed glucose transport, and increased activity of the proinflammatory factors [14] that contribute to increased insulin resistance [31]. Anxiety and depression accompanying DT2 cause patients to neglect regularly taking medications, regularly controlling glycemia, properly dieting, and regularly engaging in physical activity. The above listed neglect hamper metabolic compensations and accelerate chronic complications including atherosclerosis [32, 33]. The above listed malfunctions in DT2 impede everyday functioning and may induce or intensify anxiety and depression [34]. Pathogenically, DT2, depression, and atherosclerosis may be connected with chronic inflammation [35, 36] (Figure 8).

It was reported that depression in DT2 significantly increased risk of cardiovascular death [41]. The atherosclerosis risk is evaluated by the determination of serum lipidogram parameters: total cholesterol, HDL cholesterol, LDL cholesterol, and triacylglycerols. Unfortunately, only serum total cholesterol has been routinely determined, resulting in a deficient picture of the vascular system. In our research, the serum concentration of total cholesterol was an unsuitable marker of atheromatosis risk in DT2 patients. The only remaining elements of lipidogram (serum HDL cholesterol, LDL cholesterol, and total triacylglycerols) and Castelligro atherogenic index suggested increased risk of atherosclerosis in the group of DT2 patients in comparison to the control group. It should be mentioned that DT2 patients are routinely treated with drugs lowering concentrations of serum lipidogram parameters, but not necessarily lowering the atheromatic complications. We propose the determination of HEX in serum of DT2 patients because HEX and other lysosomal exoglycosidases are markers of tissue degradation and remodeling [42], inflammation, and atherosclerosis. In DT2 patients, serum HEX activity positively correlated with serum LDL cholesterol, serum triacylglycerols, and Castelligro atherogenic index. Therefore, serum HEX activity in DT2 patients seems to be definitely a better marker of atherosclerosis risk than serum total cholesterol. Our results demonstrate a significant increase in the anxiety and depression of DT2 patients in comparison to the control group. This suggests that testing DT2 patients in the direction of anxiety and depression is useful and creates the possibility for earlier detection of affective disorders accompanying DT2. Early detection of affective disorders creates the possibility for their compensation, better prognosis concerning quality of life, and longevity of the DT2 patients.

## 5. Conclusion

Serum HEX activity is a better marker of atherosclerosis risk in DT2 patients with mild levels of depression and anxiety than serum cholesterol.

## Figures and Tables

**Table 1:** Correlations in DT2 patients between serum HEX and level of anxiety, depression, and parameters of atherosclerosis risk (^∗^*p* < 0.05; ^∗∗^*p* < 0.01).

DT2	Spearman (*r*_s_)	*t* (*n*−2)	*p*
Anxiety level and HEX	−0.19	−1.18	0.25
Depression level and HEX	−0.16	−1.02	0.31
Cholesterol and HEX	0.22	1.24	0.16
TAG and HEX	**0.32**	**2.06**	**0.046** ^∗^
LDL cholesterol and HEX	**0.37**	**2.46**	**0.02** ^∗^
HDL cholesterol and HEX	−0.31	−1.99	0.05
Castelligro atherogenic index and HEX	**0.41**	**2.80**	**0.008** ^∗∗^

## Data Availability

No data were used to support this study.
